# Cerebellar Tuberculoma as a Rare Manifestation of Central Nervous System Tuberculosis: Literature Review and Clinical Case

**DOI:** 10.3390/diagnostics16091326

**Published:** 2026-04-28

**Authors:** Anna Starshinova, Irina Dovgalyuk, Anastasia Kulpina, Dmitry Kudlay, Olga Rasmologova, Lubov Mitrofanova

**Affiliations:** 1 Department of Mathematics and Computer Science, Saint Petersburg State University, 199034 Saint Petersburg, Russia; 2 Department of Medicine, Almazov National Medical Research Center of the Ministry of Health of the Russian Federation, 197341 Saint Petersburg, Russia; 3 Department of Pharmacology, Institute of Pharmacy, Sechenov University, 119002 Moscow, Russia; 4 NRC Institute of Immunology FMBA of Russia, 115522 Moscow, Russia; 5 Faculty of Bioengineering and Bioinformatics, Lomonosov Moscow State University, 119991 Moscow, Russia

**Keywords:** central nervous system tuberculosis, diagnostic, cerebellum, histological images, immunological tests

## Abstract

Central nervous system tuberculosis (CNS-TB) is a severe extrapulmonary manifestation of tuberculosis associated with high morbidity and mortality, particularly in children. While tuberculous meningitis remains the predominant form, focal parenchymal lesions such as tuberculomas and abscesses are less common. Cerebellar tuberculomas represent an exceptionally rare localization and may mimic posterior fossa tumors, leading to diagnostic delay. This article provides an updated review of CNS tuberculosis with special emphasis on pediatric epidemiology and cerebellar involvement, and presents a clinical case of surgically treated cerebellar tuberculoma. This clinical case demonstrates the difficulties in differential diagnosis of cerebellar tuberculosis from a tumor in this location.

## 1. Global Burden of Pediatric Tuberculosis

Tuberculosis (TB) remains a major cause of morbidity and mortality among children worldwide. According to recent World Health Organization (WHO) estimates, approximately 1.25 million children under 15 years of age developed active tuberculosis globally in 2023, accounting for nearly 12% of all reported TB cases [1,2]. Despite advances in diagnostics and treatment, childhood TB remains substantially underdiagnosed, with up to 50% of pediatric cases either unrecognized or not reported, particularly in low- and middle-income countries [1]. TB-related mortality among children is disproportionately high. It is estimated that over 190,000 children die annually from tuberculosis, with the majority of deaths occurring in children younger than five years of age and in those who did not receive timely anti-tuberculosis therapy [3,4].

Tuberculosis remains a significant public health concern in the Russian Federation, despite a gradual decline in incidence over recent years. The Russian Federation (RF) finds itself in a different situation. In 2022, the country was removed from the list of countries with a high tuberculosis burden. The tuberculosis incidence rate in 2023 was 29.6 per 100,000 population [5]. However, according to national epidemiological data, the incidence of tuberculosis in Russia remains higher than in many European countries, with a substantial proportion of cases presenting with extrapulmonary involvement [6].

Central nervous system (CNS) tuberculosis is diagnosed in 2–5% of all tuberculosis patients and in 10% of AIDS patients suffering from tuberculosis. The incidence of CNS tuberculosis is 1–2 cases per 100,000 population per year [7,8].

Children are especially prone to severe and disseminated forms of TB due to immunological immaturity, making CNS involvement more frequent and clinically significant in this age group [9,10]. These data underscore the vulnerability of the pediatric population to severe and disseminated forms of TB.

## 2. Central Nervous System Tuberculosis in Children

Central nervous system tuberculosis (CNS-TB) represents one of the most severe manifestations of pediatric TB. CNS involvement is reported in approximately 1–10% of children with active tuberculosis, a proportion higher than that observed in adults due to immature immune responses and a greater tendency toward hematogenous dissemination in early childhood [11,12]. Among pediatric CNS-TB cases, TBM predominates, accounting for 70–80%, while focal parenchymal lesions such as tuberculomas and abscesses are less frequent but associated with a high risk of neurological sequelae [4,13]. Intracranial tuberculomas in children often occur in the context of disseminated or miliary TB, although isolated lesions in immunocompetent children have been reported. Diagnosis is particularly challenging due to nonspecific clinical presentation and radiological similarities to neoplastic or pyogenic lesions.

Focal parenchymal lesions constitute approximately 5–10% of CNS-TB cases, with the majority located supratentorially [14]. Infratentorial tuberculomas are rare and account for only 3–10% of intracranial tuberculomas, with cerebellar localization representing a small subset of these cases [15,16]. In both adults and children, cerebellar tuberculomas are considered exceptionally rare. Precise epidemiological data in the pediatric population are lacking due to the scarcity of cases; existing knowledge is derived almost exclusively from isolated case reports and small series [17,18,19].

Due to their rarity, precise epidemiological data on cerebellar tuberculomas in children are lacking, and current knowledge is derived almost exclusively from isolated case reports and small case series.

Published pediatric cases describe cerebellar tuberculomas presenting with progressive headache, cerebellar ataxia, vomiting, and signs of raised intracranial pressure, often mimicking posterior fossa tumors on neuroimaging [11,12,13,14].

## 3. Clinical Case

A 17-year-old adolescent was admitted with complaints of headache, vomiting, diplopia, and impaired coordination. Initial symptoms appeared as progressive headache and gait instability. The patient was subsequently admitted for further evaluation. Neuroimaging revealed a space-occupying lesion in the cerebellum.

From the medical history, it is known that these symptoms first appeared approximately three weeks prior to admission. Due to the presence of mass effect and diagnostic uncertainty, neurosurgical intervention was performed.

The patient was vaccinated with BCG in the maternity hospital. There was noknown contact with a patient with tuberculosis. The Diaskintest was negative in September 2025. The ELISPOT test TigraTest^®^.TB (Generium, Russia) was positive.

Neurological examination revealed signs of raised intracranial pressure and cerebellar dysfunction.

**Figure 1:**
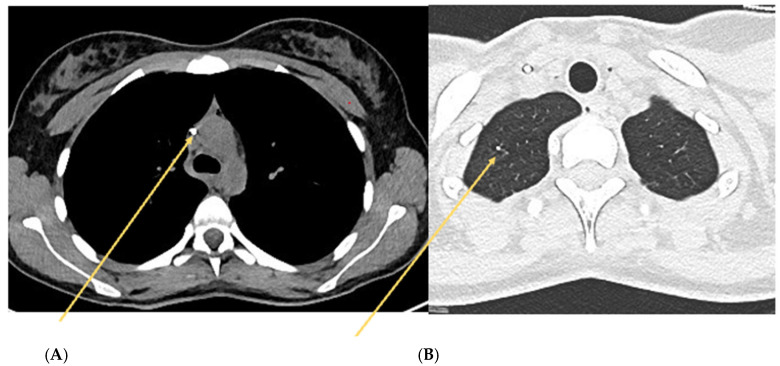
Chest CT findings demonstrating calcifications in the intrathoracic paravascular lymph nodes (**A**) and a calcification in the upper lobe of the right lung (**B**).

According to the chest CT scan dated 29 October 2025, a solitary punctate calcification measuring 1 × 1.4 mm was identified in the apical segment (S1). Calcification was also noted in the right intrathoracic bronchopulmonary lymph nodes (Figure 1).

**Figure 2:**
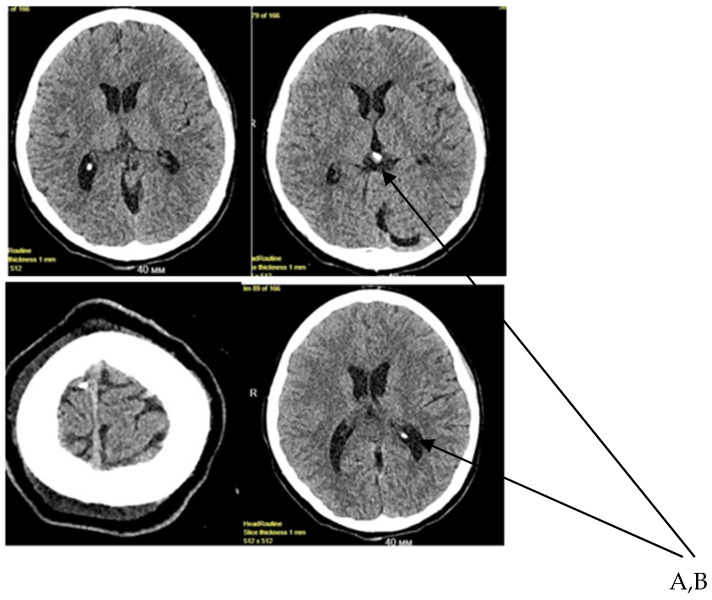
Brain MRI demonstrating a ring-enhancing lesion in the cerebellar hemisphere (arrow), associated with perifocal edema and compression of the fourth ventricle ((**A**,**B**)—calcified foci in the brain).

According to the MRI scan dated 30 September 2025, a mass lesion was identified in the cerebellum. A series of MRI scans weighted in T1 and T2 in three standard planes visualized both supra- and infratentorial brain structures (Figure 2).

### 3.1. Surgical Procedure Description

The patient was placed in the prone (Concorde) position, with the head secured in a Mayfield–Kees three-pin fixation device. The surgical field was prepared and draped in a sterile fashion, with triple antiseptic treatment. A midline skin incision was performed in the occipito-cervical region. The paraspinal muscles were dissected and retracted, and the squamous part of the occipital bone was exposed. A craniotomy was performed using a single burr hole, and a bone flap was created over the cerebellar hemispheres. The dura mater was found to be tense and was opened in a V-shaped fashion with the base directed toward the transverse sinus. Upon opening the dura, the cerebellar hemispheres protruded into the operative field. The cisterna magna was opened, resulting in the release of cerebrospinal fluid under high pressure. Gentle cerebellar retraction was performed to access the lesion. A pathological mass was visualized, appearing grayish-pink, moderately vascularized, and heterogeneous in consistency, predominantly firm, and well demarcated from the surrounding neural tissue. The lesion was removed in a stepwise manner from the right cerebellar hemisphere, vermis, and left hemisphere using microsurgical techniques, including fragmentation and bipolar coagulation. Tumor specimens were collected and sent for histopathological examination. Following resection, the cerebellum relaxed. Hemostasis was achieved. Careful inspection of the surgical cavity revealed no evidence of residual tumor tissue. Intraoperative neurophysiological monitoring remained stable throughout the procedure. The dura mater was closed in a watertight fashion. The bone flap was repositioned and secured with sutures. Layered closure of the soft tissues was performed, and a sterile dressing was applied.

#### Histological Examination of the Resected Lesion Revealed

Macroscopic description: several gray, dense tissue fragments ranging in size from 0.5 cm in diameter to 3 × 2.5 × 2 cm.

**Figure 3:**
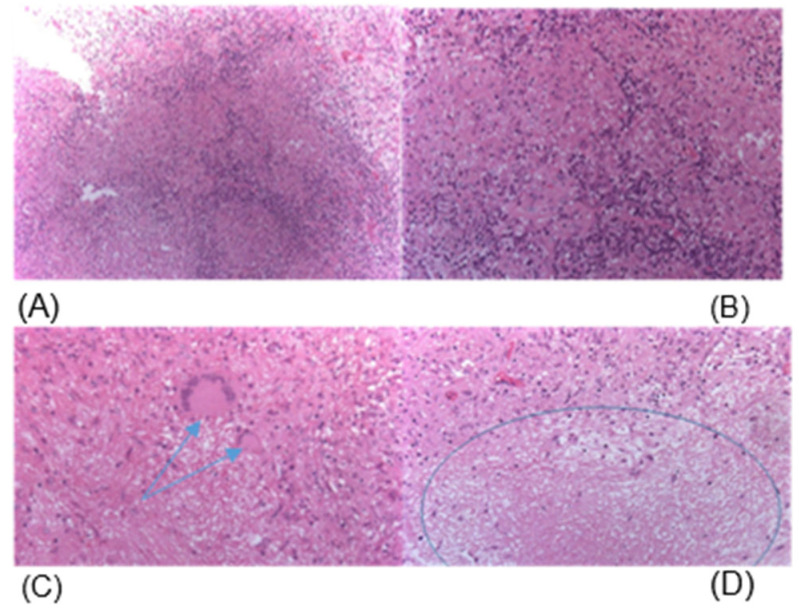
Cerebral tuberculosis. (**A**–**D**): tuberculous granulomas. (**A**)—composed of epithelioid cells surrounded by small lymphocytes (×100); (**B**)—composed of epithelioid cells surrounded by small lymphocytes (×200); (**C**)—containing multinucleated Pirogov–Langhans giant cells (indicated by arrows); (**D**)—with caseous necrosis (indicated by an oval); haematoxylin and eosin staining; ×200. Note: the background of the stained smear shows occasional unchanged erythrocytes.

Microscopic description (including additional methods of investigation): The histological specimens were reviewed collegially. The sections demonstrated features of specific granulomatous inflammation in brain tissue, represented by multiple epithelioid cell granulomas containing a small number of multinucleated giant cells of the Pirogov–Langhans type (Figure 3). The granulomas were observed at different stages of development (from necrotic to fibrotic). Ziehl–Neelsen staining did not reveal mycobacteria.

Histopathological analysis demonstrated granulomatous inflammation with caseous necrosis, consistent with tuberculoma. Immunohistochemical examination demonstrated an absence of tumor-associated marker expression (GFAP, Syn, CD117, Ki-67), diffuse fine focal expression of CD20, and absence of cytoplasmic p24 expression. Cerebrospinal fluid analysis dated 30 October 2025:

Erythrocytes: 0.00 × 10^9^/L (reference range 0.00–0.00).

Cell count (pleocytosis): 28.00 × 10^6^/L (reference range 0.00–4.00).

Microscopic examination of the stained smear:

Lymphocytes 84%, Monocytes—7%, Neutrophils—9%. Biochemical analysis:

Protein: 0.71 g/L (reference range 0.15–0.45).

## 4. Discussion and Pediatric Implications

Cerebellar tuberculoma represents a diagnostic challenge due to its nonspecific clinical presentation and radiological similarity to posterior fossa tumors. In many cases, definitive diagnosis is only established after surgical intervention and histopathological confirmation.

Tuberculosis should be considered in the differential diagnosis of all intracranial mass lesions, not only posterior fossa lesions. However, given the high burden of pediatric tuberculosis globally and the propensity for CNS involvement, tuberculosis should remain an important differential diagnosis for posterior fossa mass lesions in children, particularly in endemic regions. The presented case involves an adult patient, available pediatric data highlight that cerebellar tuberculomas can also occur in children, including immunocompetent individuals without pulmonary disease [17,18,19].

Although rare, cerebellar tuberculoma in children represents a clinically significant entity due to its potential for rapid neurological deterioration. Early recognition, prompt initiation of anti-tuberculosis therapy, and timely neurosurgical intervention when indicated are essential to achieving favorable outcomes. In TB-endemic regions, tuberculosis should remain a key diagnostic consideration in pediatric patients presenting with cerebellar mass lesions.

An integrated diagnostic approach combining clinical assessment, advanced neuroimaging, microbiological testing, and histopathological confirmation when indicated is essential for accurate diagnosis. Early identification of cerebellar tuberculoma allows for the timely initiation of anti-tuberculosis therapy and prevents irreversible neurological damage, particularly in children, who are at increased risk of severe CNS involvement and long-term sequelae.

### 4.1. Pathogenesis and Clinical Presentation

*Mycobacterium tuberculosis* (Mtb) reaches the CNS primarily via hematogenous dissemination from a pulmonary or extrapulmonary focus. Bacilli may form dormant Rich foci within the brain parenchyma, which can later evolve into granulomatous masses (tuberculomas) or liquefy to form abscesses [20].

Clinical manifestations of cerebellar tuberculoma are largely related to mass effect and the obstruction of cerebrospinal fluid pathways. Common symptoms include progressive headache, nausea and vomiting, cerebellar ataxia, gait instability, dysarthria, and signs of raised intracranial pressure or hydrocephalus [21]. In children, these symptoms may be subtle initially, contributing to delayed diagnosis.

### 4.2. Diagnostic Challenges

A structured comparison with previously reported pediatric cases demonstrates that cerebellar tuberculoma most commonly presents with signs of intracranial hypertension and cerebellar dysfunction, including ataxia and vomiting. Radiologically, these lesions frequently mimic posterior fossa tumors, particularly medulloblastoma and astrocytoma, leading to diagnostic uncertainty.

The present case highlights the importance of a multimodal diagnostic approach, integrating clinical findings, neuroimaging, microbiological studies, and histopathology. Early recognition of CNS tuberculosis is critical to ensure the timely initiation of appropriate therapy and to prevent neurological complications.

Magnetic resonance imaging is the diagnostic modality of choice. Tuberculomas typically appear as ring-enhancing lesions with surrounding vasogenic edema, sometimes showing a hypointense core on T2-weighted images corresponding to caseous necrosis [22]. MRI is the imaging modality of choice for cerebellar tuberculoma. Typical findings include well-defined intra-axial lesions with ring or nodular contrast enhancement, surrounding vasogenic edema, and mass effect on the fourth ventricle, frequently leading to obstructive hydrocephalus [14,22,23].

Solid caseating tuberculomas often appear hypointense on T2-weighted images, a feature that may help distinguish them from neoplastic lesions [21,24,25].

Advanced MRI techniques provide additional diagnostic value:

- Magnetic resonance spectroscopy (MRS) commonly demonstrates a prominent lipid peak, reflecting caseous necrosis, which favors tuberculoma over high-grade tumors [26,27].
- Diffusion-weighted imaging (DWI) may show restricted diffusion; however, this is typically less intense than in pyogenic abscesses, assisting in differential diagnosis [28].

Computed Tomography (CT) may demonstrate hyperdense or isodense cerebellar lesions with ring enhancement and perifocal edema. Although less specific than MRI, CT is valuable for the rapid detection of hydrocephalus and acute intracranial hypertension, particularly in emergency settings [22].

However, imaging findings are nonspecific and frequently mimic posterior fossa tumors, metastases, or pyogenic abscesses. Consequently, histopathological examination remains the gold standard for definitive diagnosis, demonstrating granulomatous inflammation with epithelioid cells, Langhans giant cells, and central caseous necrosis, with or without acid-fast bacilli [19].

Magnetic resonance imaging typically demonstrates ring-enhancing lesions with perifocal edema, findings that are indistinguishable from neoplastic or pyogenic processes without histopathological confirmation.

Because cerebellar tuberculoma may occur without pulmonary involvement, chest radiography or computed tomography may be normal. Nevertheless, systematic screening for extracranial TB is recommended and includes chest imaging, tuberculin skin testing or interferon-gamma release assays (IGRA), and evaluation for lymph node or skeletal involvement, particularly in children.

In isolated cerebellar tuberculomas without meningeal involvement, cerebrospinal fluid (CSF) findings are frequently normal or nonspecific. When abnormalities are present, they may include mild lymphocytic pleocytosis and elevated protein levels [24,29].

Conventional microbiological methods, including Ziehl–Neelsen staining and mycobacterial culture of CSF, have low sensitivity in parenchymal CNS tuberculosis. Molecular assays such as Xpert MTB/RIF and Xpert MTB/RIF Ultrahave improved diagnostic yield, although their sensitivity remains higher in tuberculous meningitis than in isolated tuberculomas [30,31]. Microbiological confirmation of CNS tuberculosis remains challenging. Ziehl–Neelsen staining demonstrates low sensitivity due to the paucibacillary nature of intracranial lesions, which explains the high rate of negative results reported across case series. In most reported cases, microbiological confirmation is limited due to the paucibacillary nature of CNS tuberculosis. Ziehl–Neelsen staining is often negative, with reported sensitivity below 20–30% in CNS specimens. Similarly, tuberculin skin tests may yield false-negative results in children with severe or disseminated disease [7,8,9].

Nucleic acid amplification tests, such as Xpert MTB/RIF and Xpert MTB/RIF Ultra, have improved diagnostic yield in CNS tuberculosis. Although sensitivity remains higher in tuberculous meningitis than in isolated tuberculomas, these assays are valuable when positive and can provide rapid information on rifampicin resistance.

In contrast, interferon-γ release assays, including ELISPOT, have demonstrated higher sensitivity in extrapulmonary tuberculosis and may provide supportive evidence in diagnostically challenging cases. However, these assays cannot distinguish between latent and active infection and should be interpreted in conjunction with clinical and radiological findings [6]. Interestingly, even in surgically confirmed cases, immunological tests may yield discordant results, as demonstrated by reports of negative IGRA despite histologically confirmed tuberculoma. This finding underscores the need for multimodal diagnostic approaches. Our case is consistent with the literature in terms of clinical presentation and imaging features but is notable for the discordance between negative conventional microbiological tests and a positive ELISPOT result. This finding underscores the potential adjunctive role of immunological assays in CNS tube.

In isolated cerebellar tuberculomas without meningeal involvement, CSF findings are often normal or nonspecific. When abnormalities are present, they may include mild lymphocytic pleocytosis, elevated protein, and normal or mildly reduced glucose levels.

Histopathological examination remains the gold standard for definitive diagnosis. Surgical biopsy or excision typically reveals granulomatous inflammation with epithelioid histiocytes, Langhans-type multinucleated giant cells, and central caseous necrosis. Acid-fast bacilli may or may not be demonstrable [16,19].

Histopathological examination remains the gold standard for definitive diagnosis. Surgical biopsy or excision typically reveals:

- Granulomatous inflammation with epithelioid histiocytes;
- Langhans-type multinucleated giant cells;
- Central caseous necrosis;
- Variable detection of acid-fast bacilli on Ziehl–Neelsen staining.

Histological confirmation is particularly important when imaging findings are atypical, when there is no response to empirical anti-tuberculosis therapy, or when malignancy cannot be excluded [21,32].

Microbiological confirmation of *Mycobacterium tuberculosis* from CSF is limited by low sensitivity. Conventional Ziehl–Neelsen staining and culture are frequently negative, particularly in parenchymal disease.

Although anti-tuberculosis therapy remains the cornerstone of treatment, surgical intervention is frequently required in cerebellar lesions due to mass effect in the posterior fossa, obstructive hydrocephalus, and diagnostic uncertainty (tumor vs. infection).

In the case reported by Koipapi et al., a combination of ventriculoperitoneal shunting and surgical resection was necessary due to hydrocephalus [17]. Similarly, other reports confirm that surgical resection is both diagnostic and therapeutic, particularly in cases with unclear etiology or severe mass effect.

Several reported pediatric cases required surgical intervention due to obstructive hydrocephalus, mass effect, or diagnostic uncertainty. Histopathological examination consistently revealed granulomatous inflammation with Langhans giant cells and central caseous necrosis, confirming tuberculous etiology [9,10]. These observations highlight the importance of considering tuberculosis in the differential diagnosis of posterior fossa masses in children, even in the absence of pulmonary disease or immunodeficiency.

### 4.3. Medical Management

Standard anti-tuberculosis therapy, including isoniazid, rifampicin, pyrazinamide, and ethambutol, remains the cornerstone of treatment and is typically administered for 9–12 months or longer, depending on clinical and radiological response [20]. Corticosteroids are frequently used as adjunctive therapy to reduce cerebral edema and intracranial pressure.

### 4.4. Role of Surgery

Surgical intervention is reserved for selected cases and is indicated in the presence of life-threatening intracranial hypertension, progressive neurological deterioration despite adequate medical therapy, paradoxical enlargement of lesions, or diagnostic uncertainty requiring tissue confirmation [21]. In published series of CNS tuberculomas, approximately 50–60% of patients underwent surgical intervention, with favorable outcomes when combined with anti-tuberculosis therapy [22].

## 5. Conclusions

Cerebellar tuberculoma represents a diagnostic challenge due to its nonspecific clinical presentation and radiological similarity to posterior fossa tumors. In many cases, definitive diagnosis is only established after surgical intervention and histopathological confirmation. The present case highlights the importance of a multimodal diagnostic approach, integrating clinical findings, neuroimaging, microbiological studies, and histopathology. A multimodal diagnostic approach integrating neuroimaging, immunological assays, and histopathology is essential. Early recognition of CNS tuberculosis is critical to ensure the timely initiation of appropriate therapy and to prevent neurological complications [33,34]. Surgical intervention should be considered in cases with significant mass effect or diagnostic uncertainty.

The presented case of a 17-year-old adolescent with a cerebellar mass demonstrates the diagnostic complexity of central nervous system tuberculosis. Despite negative Ziehl–Neelsen staining and a negative ATP skin test, histological findings were consistent with cerebellar tuberculoma, and an ELISPOT (TigraTest)-based immunological assay was positive, supporting tuberculosis infection. This case highlights that focal CNS tuberculosis may mimic neoplastic processes and often lacks microbiological confirmation. It underscores the limited sensitivity of conventional skin testing and the important diagnostic value of modern immunological methods, particularly interferon-γ release assays [35,36]. Systematic screening for tuberculosis infection using immunological assays should therefore be considered in children and adolescents with unexplained intracranial lesions or clinical suspicion of extrapulmonary TB [37,38,39], in order to ensure timely diagnosis and initiation of appropriate therapy.

## Data Availability

Not applicable. No new data were created or analyzed in this study.
